# Increased DNA Dicarbonyl Glycation and Oxidation Markers in Patients with Type 2 Diabetes and Link to Diabetic Nephropathy

**DOI:** 10.1155/2015/915486

**Published:** 2015-04-09

**Authors:** Sahar Waris, Brigitte M. Winklhofer-Roob, Johannes M. Roob, Sebastian Fuchs, Harald Sourij, Naila Rabbani, Paul J. Thornalley

**Affiliations:** ^1^Warwick Medical School, Clinical Sciences Research Laboratories, University of Warwick, University Hospital, Coventry CV2 2DX, UK; ^2^Human Nutrition & Metabolism Research and Training Center Graz, Institute of Molecular Biosciences, Karl Franzens University, 8010 Graz, Austria; ^3^Clinical Division of Nephrology, Department of Internal Medicine, Medical University of Graz, 8036 Graz, Austria; ^4^Clinical Division of Endocrinology and Nuclear Medicine, Department of Internal Medicine, Medical University of Graz, 8036 Graz, Austria; ^5^Warwick Systems Biology Centre, Senate House, University of Warwick, Coventry CV4 7AL, UK

## Abstract

*Aim*. The aim of this study was to assess the changes of markers of DNA damage by glycation and oxidation in patients with type 2 diabetes and the association with diabetic nephropathy. *Methodology*. DNA oxidation and glycation adducts were analysed in plasma and urine by stable isotopic dilution analysis liquid chromatography-tandem mass spectrometry. DNA markers analysed were as follows: the oxidation adduct 7,8-dihydro-8-oxo-2′-deoxyguanosine (8-OxodG) and glycation adducts of glyoxal and methylglyoxal—imidazopurinones GdG, MGdG, and N_2_-(1,*R/S*-carboxyethyl)deoxyguanosine (CEdG). *Results*. Plasma 8-OxodG and GdG were increased 2-fold and 6-fold, respectively, in patients with type 2 diabetes, with respect to healthy volunteers. Median urinary excretion rates of 8-OxodG, GdG, MGdG, and CEdG were increased 28-fold, 10-fold, 2-fold, and 2-fold, respectively, in patients with type 2 diabetes with respect to healthy controls. In patients with type 2 diabetes, nephropathy was associated with increased plasma 8-OxodG and increased urinary GdG and CEdG. In a multiple logistic regression model for diabetic nephropathy, diabetic nephropathy was linked to systolic blood pressure and urinary CEdG. *Conclusion*. DNA oxidative and glycation damage-derived nucleoside adducts are increased in plasma and urine of patients with type 2 diabetes and further increased in patients with diabetic nephropathy.

## 1. Introduction

**Figure 1:**
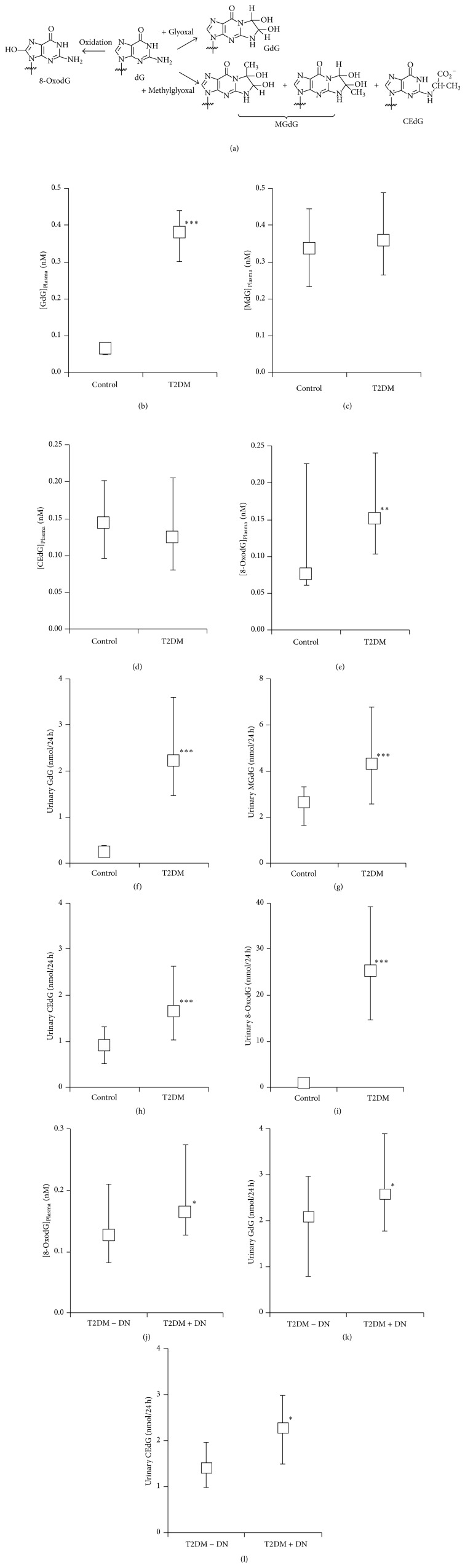
(a) Formation of glycation and oxidation adducts of deoxyguanosine. The common 2′-deoxyribosyl group has been omitted for clarity. (b)–(i) Nucleotide glycation and oxidation adducts in plasma and urine of healthy controls subjects and patients with type 2 diabetes. Plasma: (b) GdG, (c) MGdG, (d) CEdG, and (e) 8-OxodG. Urine: (f) GdG, (g) MGdG, (h) CEdG, and (i) 8-OxodG. (j)–(l) Nucleotide glycation and oxidation adducts in plasma and urine in patients with type 2 diabetes with and without nephropathy. (j) Plasma 8-OxodG, (k) urinary GdG, and (l) urinary CE-dG. Data are median with the upper and lower quartile as error bars. Significance: ^∗^ *P* < 0.05, ^∗∗^ *P* < 0.01, and ^∗∗∗^ *P* < 0.001; Mann-Whitney *U*.

Reactive oxygen species (ROS) and dicarbonyl metabolites, glyoxal and methylglyoxal, are some of the most reactive metabolites present in human metabolism. They are increased in diabetes and diabetic nephropathy [1–3]. ROS react with deoxyguanosine residues of DNA to form 8-dihydro-8-oxo-2′-deoxyguanosine (8-OxodG). The DNA content of 8-OxodG residues was increased in lymphocyte DNA in clinical diabetes [4]. Reactive dicarbonyl intermediates of endogenous glycation, glyoxal and methylglyoxal, also react with deoxyguanosine residues of DNA to form mainly imidazopurinone adducts. Glyoxal forms 3-(2′-deoxyribosyl)-6,7-dihydro-6,7-dihydroxyimidazo-[2,3-b]purin-9(8)one (GdG) and methylglyoxal forms two imidazopurinone structural isomers, 3-(2′-deoxyribosyl)-6,7-dihydro-6,7-dihydroxy-6/7-methylimidazo-[2,3-b]purine-9(8)one (MGdG). DNA glycation by methylglyoxal also forms two stereoisomers, N_2_-modified derivatives, N_2_-(1,*R/S*-carboxyethyl)-deoxyguanosine (CEdG) [5], Figure 1(a). GdG, MGdG, and CEdG are nucleotide advanced glycation endproducts (AGEs). Methylglyoxal-derived MGdG was a quantitatively major adduct of endogenous damage in healthy volunteers [5]. Damage to DNA by endogenous glyoxal and methylglyoxal is suppressed by glyoxalase 1 (Glo1) of the cytoplasmic glyoxalase system [6]. The expression and activity of Glo1 in the kidney were decreased in experimental models of diabetic nephropathy—streptozotocin-induced diabetes in mice and rats and the db/db diabetic mouse [7–9]. This is associated with increased dicarbonyl glycation of proteins linked to the development of diabetic nephropathy [10]. Increased urinary 8-OxodG excretion has been found in patients with diabetes type 2 diabetes (T2DM), with further increases associated with the presence of microvascular complications, and was a predictor of diabetic nephropathy [11, 12].

Nucleotide AGEs are associated with DNA single-strand breaks and increased mutation frequencies [13]. Oxidised and glycated nucleosides are removed from DNA by nucleotide excision repair of DNA damaged by oxidation and glycation. Recent functional genomic studies of Glo1 have linked dicarbonyl glycation to the development of diabetic nephropathy [14, 15]. The plasma concentration and urinary fluxes of glyoxal and methylglyoxal-derived nucleoside AGEs in diabetes and diabetic nephropathy are unknown.

Reliable quantitation of oxidised and glycated nucleosides has proven difficult because of inadequate sensitivity and specificity of detection responses and poor adduct stability and recovery during preanalytic processing. Initial studies involved ^32^P-labelling studies of DNA digests [16] and immunoassay [17]. Stable isotopic dilution analysis liquid chromatography with tandem mass spectrometric detection (LC-MS/MS) has been used for assay of 8-OxodG [18]. For estimation of both DNA glycation and oxidation adducts, the high specificity and sensitivity of LC-MS/MS makes this the preferred method for DNA damage marker analysis. We developed a stable isotopic dilution analysis LC-MS/MS method to concurrently quantify GdG, MGdG, CEdG, and 8-OxodG in plasma and urinary ultrafiltrate [5]. 8-OxodG has been studied extensively in plasma and urine as a biomarker of oxidative damage [18] whereas the diagnostic utility of DNA glycation adducts is unknown.

Herein we analysed DNA oxidation and glycation markers in plasma and urine of patients with T2DM with and without diabetic nephropathy. Healthy volunteers served as controls. The outcome revealed marked increases in DNA damage in T2DM and further increase of selected markers in diabetic nephropathy. Increased urinary excretion of glycation adducts GdG and CEdG was indicative of diabetic nephropathy.

## 2. Methods

### 2.1. Participants

Patients with type 2 diabetes and overt nephropathy (T2DM + DN) and patients with T2DM and without overt nephropathy (T2DM − DN) were recruited in the EU-funded PREDICTIONS project [19]. Healthy volunteers were recruited in the EU-funded VITAGE project [20]. Both studies were conducted at the Human Nutrition and Metabolism Research and Training Center, Karl Franzens University of Graz, Austria, and the Clinical Division of Nephrology and the Clinical Division of Endocrinology and Nuclear Medicine, Department of Internal Medicine, Medical University of Graz, Austria. The study protocols were approved by the Ethics Committee of the Medical University of Graz, Austria, and written informed consent was obtained from all study subjects. Diagnosis of diabetes was established in accordance with the WHO criteria: fasting plasma glucose ≥7.0 mmol/L, a two-hour value in an oral glucose tolerance test 11.1 mmol/L, or random plasma glucose 11.1 mmol/L in the presence of symptoms, aged 35–75 with a documented duration of diabetes of ≥5 years being eligible. T2DM was diagnosed by lack of criteria for type 1 diabetes. Inclusion criteria for cases were albuminuria >300 mg/d and known overt diabetic retinopathy. Retinopathy was requested to be present to ensure that albuminuria is the consequence of diabetic nephropathy rather than a nondiabetic glomerulopathy. A renal biopsy would be the gold standard to discriminate between diabetic nephropathy and a nondiabetic glomerulopathy, but a renal biopsy is rarely taken in patients with T2DM. Several studies have indicated that presence of retinopathy is a good alternative inclusion criterion to discriminate between diabetic nephropathy and nondiabetic glomerulopathy in patients with T2DM with albuminuria [21–23]. Exclusion criteria were chronic renal failure, known causes of renal failure other than diabetes, and non-Caucasian ethnic origin. Cases and controls were matched for gender and diabetes duration. Exclusion criteria for T2DM-DN were microalbuminuria, non-Caucasian ethnic origin, and, in case of use of RAAS-blocking medication, unknown albuminuria status prior to start of treatment. Estimated GFR (eGFR) was determined by the Chronic Kidney Disease Epidemiology Collaboration equations: for females (for whom [Creatinine]_plasma_ was >62 *μ*M), eGFR = 144 × ([Creatinine]_plasma_/62)^−1.209^ × (0.993)^Age^; and, for males, when [Creatinine]_plasma_ ≤ 80 *μ*M, eGFR = 141 × ([Creatinine]_plasma_/80)^−0.411^ × (0.993)^Age^, and when [Creatinine]_plasma_ > 80 *μ*M, eGFR = 141 × ([Creatinine]_plasma_/80)^−1.209^ × (0.993)^Age^ [24]. Glycated haemoglobin (A1C) was determined by a validated ion exchange high pressure chromatography method (Menarini ARKRAY ADAMS A1C HA-8180 analyser, Menarini Diagnostics, Florence, Italy) [25]. Subject characteristics are given in Table 1.

### 2.2. Methods

Venous blood samples were collected from all study subjects after overnight fast and plasma samples were prepared immediately and stored at −80°C until analysis; 24 h urines collections were made and aliquots prepared and stored at −80°C until analysis. Ultrafiltrates were prepared by microspin ultrafiltration (10 kDa cut-off) of plasma and urine (100 *μ*L), collecting* ca.* 50 *μ*L ultrafiltrate. Sample processing and storage validation were published previously [5].

Nucleotide markers of glycation and oxidation were determined by stable isotopic dilution analysis LC-MS/MS. GdG, MGdG, CEdG, and 8-OxodG and related stable isotopic [^13^C_10_, ^15^N_5_] substituted standards were prepared as described [5]. For LC-MS/MS, plasma and urine ultrafiltrates (40 *μ*L) were spiked with 10 *μ*L isotopic standard mixture containing 0.1 nmol [^13^C_10_, ^15^N_5_] dG, 1 pmol [^13^C_10_, ^15^N_5_] 8-OxodG, 1 pmol [^13^C_10_, ^15^N_5_] MGdG and CEdG, and 1 pmol [^13^C_10_, ^15^N_5_] GdG. LC-MS/MS was performed using an Acquity UPLC-Quattro Premier tandem mass spectrometer with a BEH C18 1.7 *μ*m particle size, 2.1 × 100 mm column. The mobile phase (0.25 mL/min) was 0.1% formic acid with a linear gradient of 0–10% acetonitrile from 2 to 10 min and isocratic 10% acetonitrile from 10 to 15 min. After analysis, the column was washed with 50% acetonitrile, 0.1% formic acid for 10 min and thereafter reequilibrated with initial mobile phase for 10 min. The column temperature was varied from 10°C. For GdG, MGdG, CEdG, and 8-OxodG, limits of detection were 0.8, 2.5, 2.2, and 0.7 fmol; analytical recoveries were 104, 97, 98%, and 99%, respectively, and coefficients of variation 2–7% [5].

### 2.3. Statistical Analysis

Data are median (minimum–maximum) or median (lower–upper quartile) values. Significance of differences between means was assessed by Mann-Whitney *U* test. Bivariate regression was nonparametric (Spearman) and logistic regression was performed of DN on continuous variables, excluding the recruitment qualifier of urinary albumin, solving for regression coefficient *B*. Statistical analysis was performed by the SPSS software, v21.

## 3. Results

### 3.1. DNA Glycation Adducts in Plasma and Urine

Biomarkers of nucleotide glycation and oxidation are conveniently determined by assay of glycated and oxidised nucleosides in plasma and urine ultrafiltrates. This was performed with samples from healthy controls and patients with T2DM with and without diabetic nephropathy. Median plasma concentrations of GdG, MGdG, CEdG, and 8-OxodG in healthy subjects were 0.07, 0.34, 0.14, and 0.08 nM, respectively. Plasma GdG was increased 6-fold and plasma 8-OxodG increased 2-fold in patients with T2DM. Median urinary excretion rates of GdG, MGdG, CEdG, and 8-OxodG in healthy subjects were 0.23, 2.63, 0.90, and 0.90 nmol/24 h, respectively. In patients with T2DM, urinary GdG, MGdG, CEdG, and 8-OxodG were increased 10-fold, 2-fold, 2-fold, and 28-fold, respectively, Figures 1(b)–1(i).

We also assessed the effect of diabetic nephropathy (DN) in patients with T2DM on plasma and urinary nucleotide glycation and oxidation damage markers. In plasma, median 8-OxodG concentration was increased 30% in DN and in urine GdG was increased 24% and CEdG increased 60% in diabetic nephropathy, Figures 1(j)–1(l).

### 3.2. Correlation Analysis

Bivariate correlation analysis of clinical chemistry variables of renal function with nucleoside glycation and oxidation analytes in patients with T2DM is given in Table 2. As expected, there were a strong negative correlation of plasma creatinine with eGFR (*r* = −0.95, *P* < 0.001), a strong positive correction of plasma creatinine with urinary albumin excretion UAE (*r* = 0.61, *P* < 0.001), and a strong negative correction of eGFR with UAE (*r* = −0.55, *P* < 0.001). There were also positive correlations of urinary excretions of GdG and CEdG with UAE (*r* = 0.38 and 0.42, resp., *P* < 0.01) and a positive correlation of plasma 8-OxodG with plasma creatinine (*r* = 0.38, *P* < 0.01). For correlation between nucleotide glycation and oxidation analytes, in plasma there were a strong positive correlation of MGdG with CEdG (*r* = 0.46, *P* < 0.001) and also positive correlation of MGdG with 8-OxodG (*r* = 0.45, *P* < 0.01) and GdG with CEdG (*r* = 0.47, *P* < 0.01). For urinary excretion, there was a positive correlation of MGdG excretion with 8-OxodG excretion (*r* = 0.37, *P* < 0.01). There was also a positive correlation of plasma MGdG with urinary MGdG (*r* = 0.41, *P* < 0.01) but there were no similar correlations of plasma and urinary levels of other nucleotide glycation and oxidation analytes. There was no correlation of clinical chemistry variables of glycemic control (fasting plasma glucose, A1C), total cholesterol, LDL cholesterol, HDL cholesterol, or systolic and diastolic blood pressure with nucleotide glycation and oxidation markers.

In a multiple logistic regression analysis of diabetic nephropathy on clinical and clinical chemistry variables, the following variables were included: clinical variables previously associated with diabetic nephropathy (age, gender, A1C, systolic and diastolic blood pressure, and total cholesterol) [21, 26], related markers of metabolic control (fasting plasma glucose, LDL, and HDL), other factors linked to increased glyoxal and methylglyoxal (duration of diabetes, BMI) [1, 22], and markers of DNA glycation and oxidation measured noninvasively (urinary excretions of GdG, MGdG, CEdG, and 8-OxodG). Plasma creatinine, eGFR, and UAE were excluded from the model as established biomarkers of diabetic nephropathy. Forward stepwise selection of variables gave a multiple logistic regression model linking diabetic nephropathy to systolic blood pressure (*B* = 0.05 ± 0.02, exponent 1.05; *P* = 0.009) and urinary excretion of CEdG (*B* = 1.00 ± 0.41, exponent 2.70; *P* = 0.016, *n* = 56). This is consistent with the increased systolic blood pressure found in patients with diabetic nephropathy and the positive correlation of urinary CEdG with UAE, Tables 1 and 2.

## 4. Discussion

Herein we found, for the first time, increased plasma levels and urinary excretion of DNA glycation adducts in patients with T2DM and a link to diabetic nephropathy. Hence, measurement of DNA glycation adducts and nucleosides in body fluids may be valuable biomarkers of quantitative and functional important DNA damage* in vivo*. Glyoxal is formed in physiological systems by lipid peroxidation and degradation of glycated proteins and monosaccharides. Methylglyoxal is formed mainly by degradation of triosephosphates and also by ketone body metabolism and threonine catabolism [23]. Both dicarbonyls are metabolised by the glutathione-dependent Glo1 [6] recently linked mechanistically to the development of diabetic nephropathy [14]. Glycation of DNA by glyoxal and methylglyoxal has been linked to DNA strand breaks and mutagenesis [27, 28], and cellular dicarbonyl concentrations increase in oxidative stress [29]. Formation of MGdG and CEdG by glycation of DNA with endogenous methylglyoxal may explain the previously reported enhancement of DNA modification by glucose metabolites under conditions of glutathione depletion [30], increased DNA unwinding, and single-strand breaks of DNA in vascular endothelial cell in hyperglycemia* in vitro* [31] and increased single-strand breaks of DNA of patients with diabetes [32].

In previous studies we found levels of GdG and CEdG residues in peripheral lymphocyte DNA of healthy people to be similar to those of 8-OxodG, and the DNA content of MGdG exceeded that of the widely studied 8-OxodG [5]. Nucleotide AGE content of DNA was also markedly higher than that of other physiological aldehydes—such as 4-hydroxynonenal and malondialdehyde [33]. Modification of DNA by physiological dicarbonyls therefore gives rise to quantitatively important steady-state levels of deoxyguanosine-derived adducts in cellular DNA* in vivo*.

In patients with T2DM, there were particularly large increases in glyoxal-derived imidazopurinone GdG in plasma and urine and 8-OxodG in urine. We cannot discriminate between the contributions to glycated and oxidised nucleosides formed by repair of glycated and oxidised DNA and formed by direct glycation and oxidation of deoxyguanosine. Formation of GdG in patients with T2DM appears to be particularly favoured. For methylglyoxal-derived metabolites, CEdG is more stable chemically than MGdG and may be a more robust biomarker when released from cells [5]. LC-MS/MS analysis of urinary excretion of CEdG in streptozotocin-induced diabetic rats indicated a 4-fold increase in CEdG excretion in diabetes with respect to normal healthy control rats [34]. Increased formation of glyoxal and methylglyoxal and oxidative stress in hyperglycemia associated with diabetes has been linked to microvascular complications—including diabetic nephropathy [1, 35]. Related damage to proteins [36] and herein DNA provides potential markers of nephropathy development.

We assessed the link of urinary excretion of nucleoside glycation and oxidation adducts as potential noninvasive biomarkers of diabetic nephropathy. In multiple logistic regression analysis, urinary CEdG emerged as a positive correlate for diabetic nephropathy. Urinary CEdG also correlated positively with UAE and hence is linked to an established biomarker of diabetic nephropathy. It also likely reflects increased exposure to methylglyoxal. CEdG has higher chemical stability than MGdG and higher biological stability than methylglyoxal in cells and physiological fluids which may explain its greater diagnostic value than these related metabolites [5, 22].

Plasma and urinary 8-OxodG were also increased in patients with T2DM. Urinary oxidation adducts of RNA rather than DNA were associated with mortality in a prospective study of patient with T2DM [37]. Increased urinary 8-OxodG in patients with T2DM was found previously but the technique used, liquid chromatography with electrochemical detection, appears to have overestimated urinary levels of 8-OxodG by* ca.* 10-fold [11, 12]. The greater sensitivity and specificity for analyte detection provided by multiple reaction monitoring detection in LC-MS/MS accounts for this. The two major analytical approaches that have been used for the measurement of urinary 8-OxodG prior to application of LC-MS/MS were HPLC combined with electrochemical detection and immunoassay. Approaches other than LC-MS/MS have overestimated 8-OxodG [38–40]. Estimates of urinary 8-OxodG herein are similar to independent estimates by stable isotopic dilution analysis LC-MS/MS for healthy controls [41] and patients with T2DM [42]. Similar improvements have been made in the detection of CEdG by LC-MS/MS. CEdG was determined in urine of normal healthy human subjects previously by immunoassay with estimates in the range of 3.4–344 pmol/mg creatinine and median of* ca.* 30 pmol/mg creatinine [43]. Estimation herein by LC-MS/MS gave median (minimum–maximum) values of 0.55 (0.17–1.58) pmol/mg creatinine, suggesting that immunoassay procedures overestimated urinary CEdG by* ca.* 50-fold, as was found for similar ELISA and LC-MS/MS measurement of 8-OxodG [39]. Overestimation is likely caused by interference due to imperfect epitope specificity of the monoclonal antibody used and formation of CEdG from MGdG and other sample components during the high pH of preanalytic sample processing.

A weakness of this study was lack of females in the healthy control study group but there was no indication in the T2DM group that DNA damage marker levels were linked to gender.

## 5. Conclusion

From the quantitative amount and link to functional endpoints, GdG, MGdG, and CEdG adducts are of likely pathogenic and diagnostic significance. Recent further evidence linking dicarbonyl glycation with development of DN [14] suggests that DNA dicarbonyl adducts may emerge as biomarkers of development of DN. This study suggests that dicarbonyl adducts are not surrogate measures of metabolic control and some are linked to DN.

## Figures and Tables

**Table 1:** Characteristics of human subjects recruited for this study.

Subject group	Control	T2DM-DN	T2DM+DN
*n*	28	28	28
Age (years)	61 ± 8	63 ± 6	60 ± 10
Gender (M/F)	28/0	20/8	20/8
BMI (kg/m^2^)	26.1 ± 1.8	28.5 ± 5.0^*^	33.2 ± 5.0^∗∗∗,OOO^
Fasting plasma glucose (mM)	5.6 ± 0.5	9.4 ± 3.0^***^	9.3 ± 3.8^***^
A1C (%)	ND	7.6 ± 1.2	7.5 ± 1.5
(mmol/mol)		60 ± 13	58 ± 17
Systolic BP (mmHg)	135 ± 13	136 ± 13	153 ± 22^***^
Diastolic BP (mmHg)	84 ± 6	81 ± 10	84 ± 10
Total cholesterol (mM)	5.52 ± 0.94	5.04 ± 1.13	5.17 ± 1.14
LDL cholesterol (mM)	3.31 ± 0.69	2.92 ± 0.96	2.58 ± 0.92^*^
HDL cholesterol (mM)	1.49 ± 0.32	1.46 ± 0.38	1.40 ± 0.29
Urinary albumin (mg/24 h)	ND	13 ± 10	2437 (371–9000)^***^
eGFR (ml/min)	69 ± 13	73 ± 13	31.7 (20.0–45.3)^∗∗∗,OOO^

ND = not determined. Data are mean ± SD or median (minimum–maximum). Significance: ∗ and ∗∗∗, *P* < 0.05 and *P* < 0.001 with respect to healthy volunteers, and ^OOO^, *P* < 0.001 with respect to patients with type 2 diabetes without nephropathy.

**Table 2:** Correlation analysis of nucleotide glycation and oxidation analytes with clinical chemical variables of renal function.

Creatinine_Plasma_	—										
eGFR	−0.95^***^	—									
UAE	0.61^***^	−0.55^***^	—								
GdG_Plasma_				—							
MGdG_Plasma_					—						
CEdG_Plasma_				0.47^**^	0.46^***^	—					
8-OxodG_Plasma_	0.38^**^				0.45^**^		—				
GdG_Urine_			0.38^**^					—			
MGdG_Urine_					0.41^**^	0.39^**^			—		
CEdG_Urine_			0.42^**^						0.33^*^	—	
8-OxodG_Urine_									0.37^**^		—
Parameter	Creatinine_Plasma_	eGFR	UAE	GdG_Plasma_	MGdG_Plasma_	CEdG_Plasma_	8-OxodG_Plasma_	GdG_Urine_	MGdG_Urine_	CEdG_Urine_	8-OxodG_Urine_

Bivariate regression was by nonparametric, Spearman method. Significance: ∗∗ and ∗∗∗, *P* < 0.01 and *P* < 0.001.

## Abbreviations

A1C: Glycated haemoglobin
AGE: Advanced glycation endproduct
CEdG and CEdG_A_/CEdG_B_: N2-(1,*R/S*-Carboxyethyl)-deoxyguanosine and R/S epimers
dG: Deoxyguanosine
GdG: 3-(2′-Deoxyribosyl)-6,7-dihydro-6,7-dihydroxyimidazo-[2,3-b]purin-9(8)one
eGFR: Estimated glomerular filtration rate
Glo1: Glyoxalase 1
8-OxodG: 7,8-Dihydro-8-oxo-2′-deoxyguanosine
4HNE: 4-Hydroxynonenal
MGdG: 3-(2′-Deoxyribosyl)-6,7-dihydro-6,7-dihydroxy-6/7-methylimidazo-[2,3-b]purine-9(8)one
LC-MS/MS: Liquid chromatography with tandem mass spectrometric detection
UAE: Urinary albumin excretion
DN: Diabetic nephropathy.
